# Bottom‐up reconstitution of focal adhesion complexes

**DOI:** 10.1111/febs.16023

**Published:** 2021-05-30

**Authors:** Stephanie Schumacher, Roberto Vazquez Nunez, Christian Biertümpfel, Naoko Mizuno

**Affiliations:** ^1^ Department of Structural Cell Biology Max Planck Institute of Biochemistry Martinsried Germany; ^2^ Laboratory of Structural Cell Biology National Heart, Lung, and Blood Institute National Institutes of Health Bethesda MD USA; ^3^ National Institute of Arthritis and Musculoskeletal and Skin Diseases National Institutes of Health Bethesda MD USA

**Keywords:** actin, integrin, PIP2, talin, vinculin

## Abstract

Focal adhesions (FA) are large macromolecular assemblies relevant for various cellular and pathological events such as migration, polarization, and metastatic cancer formation. At FA sites at the migrating periphery of a cell, hundreds of players gather and form a network to respond to extra cellular stimuli transmitted by the integrin receptor, the most upstream component within a cell, initiating the FA signaling pathway. Numerous cellular experiments have been performed to understand the FA architecture and functions; however, their intricate network formation hampers unraveling the precise molecular actions of individual players. Here, *in vitro* bottom‐up reconstitution presents an advantageous approach for elucidating the FA machinery and the hierarchical crosstalk of involved cellular players.

## Graphical abstract

Integrin acts as the master receptor to initiate the large focal adhesion assembly. The activation of integrin is mediated by both extracellular ligands and the intracellular switches talin and kindlin. The elucidation of the precise molecular mechanism of the bidirectional integrin activation is crucial for picturing a comprehensive view of cellular adhesion. Bottom‐up reconstitution is an invaluable tool to study the assembly process.

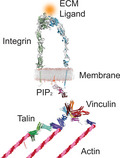

**Abbreviations**
ECM: extracellular matrix
FAs: Focal adhesions
NMR: Nuclear magnetic resonance
PIP2: phosphatidylinositol‐4,5‐bisphosphate
TM: transmembrane

Cellular adhesions are crucial for the development of multicellular organisms and tissue morphogenesis, as they enable cells to connect to each other and to their environment. Cells attach to their surroundings via large intracellular molecular assemblies called focal adhesions (FA), which are localized at the plasma membrane. They are responsible for two major cellular processes: First, they provide direct mechanical links between the extracellular matrix (ECM) and the cell through connections to the cytoskeleton. Second, they sense the environment and bidirectionally transmit signals across the plasma membrane [1, 2, 3]. Besides providing structural connections, FAs also play a role in transmitting signals that can affect cell survival or differentiation by altering gene expression (reviewed in [4]). Aberrant FA functions have detrimental effects and are linked to several pathologies. Therefore, it is critical to understand how cells manage to properly function during adhesion and how they dynamically connect to their surrounding tissue.

A central component of FAs is the transmembrane integrin receptor, recognizing extracellular cues [5]. The activation of integrin can be triggered either by the binding of an extracellular ligand like fibronectin (outside‐in signaling) or by intracellular proteins talin and kindlin (inside‐out signaling) [5]; therefore, it transmits bi‐directional signals. In both signaling directions, activated integrin further facilitates the recruitment of FA players and the FAs mature into a vast complex containing ~200 different proteins that extensively connect to the actin cytoskeleton [2, 6, 7]. Using super‐resolution microscopy techniques [8, 9, 10], organizational properties of adhesions, both in vertical (perpendicular to plasma membrane) and lateral (parallel to membrane) directions, have been studied. 3D imaging revealed that a ~ 40‐nm‐wide region lies between integrins and the actin cytoskeleton, which consists of three layers: the integrin signaling, the force transduction, and the actin regulatory layers [9] (Fig. 1). Each layer contains distinct sets of proteins, with an exception of talin that connects between integrin and actin, thus, spanning over all three layers [11]. Laterally, FAs extend along the actin retrograde flow as maturation proceeds, and proteins such as talin and vinculin align in this direction [12, 13, 14].

**Fig. 1:**
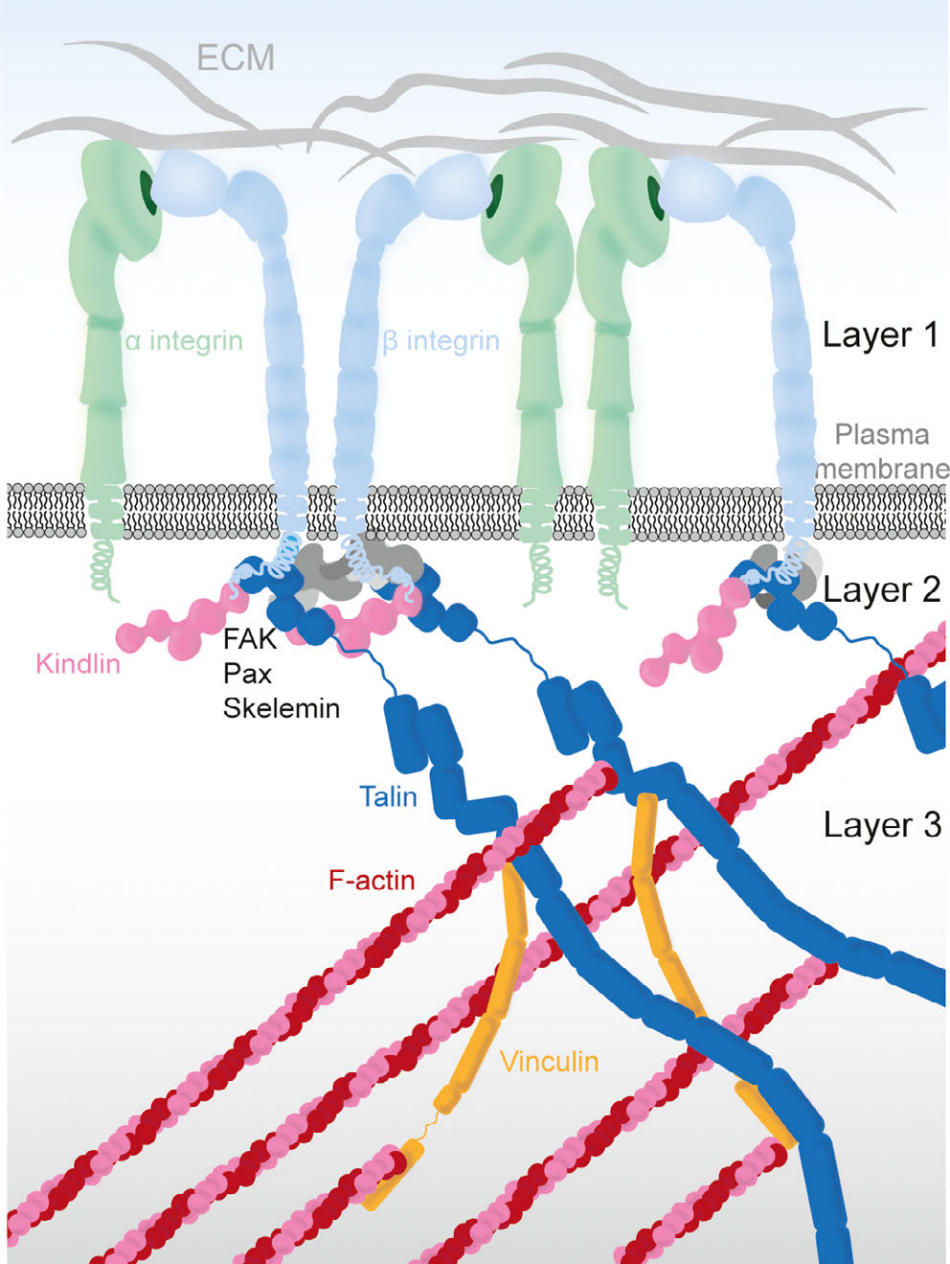
Simplified schematic of the FA machinery focusing on the activation of integrin. Components of the ECM (gray) bind to integrin receptors (α subunit in light green and β subunit in light blue), which reach through the plasma membrane (dark gray) into the cytosol. Intracellular proteins kindlin (pink) and talin (blue) bind to the cytoplasmic tail of β‐integrin together with additional signaling factors like FAK, Pax and Skelemin (gray). Activated talin extends through all FA layers from the integrin receptor to the actin cytoskeleton (red/pink) and vinculin (dark yellow) enforces the talin‐actin interaction. The FA machinery is tightly regulated and allows bidirectional signal transduction from outside‐in and from inside‐out.

The mechanism of interactions of the key proteins with integrin, actin fibers, and other FA proteins has been extensively studied, employing numerous techniques such as biophysical techniques, mass spectrometry, super‐resolution, and structural biology methods like electron microscopy, X‐ray crystallography, or NMR [9, 15, 16, 17, 18]. However, the complicated FA network prevents us from understanding the precise molecular functions of the FA components. This is because individual components are part of various submodules within FAs and their functions cannot be easily dissected within the synergistic network or with only truncated domains lacking full regulatory functions. Therefore, building up submachineries of FAs from individual, fully regulatable components, gives a great advantage to elucidate the hierarchical interactions of FA players. This ‘bottom‐up’ reconstitution allows to systematically connect different functional subcomplexes toward a comprehensive understanding of FAs.

In this review, we will focus on the efforts of such bottom‐up reconstitutions, particularly from the FA initiation process to the attachment of actin bundles onto the plasma membrane (Fig. 1). We will discuss the recent findings on the molecular crosstalk toward FA network formation. The elucidation of the fundamental molecular mechanisms of these players will lead to a general understanding of how cells attach and react to their surroundings, which is the first step in comprehending how aberrations in these processes contribute to diseases such as cancer [2].

## FA core initiation machinery

### Integrins

**Fig. 2:**
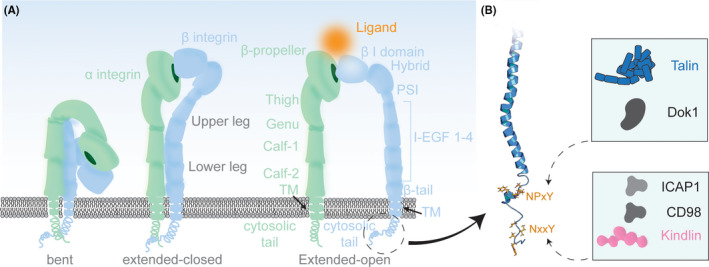
Domain architecture of integrin receptors. (A) α‐integrins consist of a β‐propeller head, thigh, Genu, Calf‐1, calf‐2, TM helix, and cytoplasmic tail domains. β‐integrins comprise a βI head, hybrid, PSI, I‐EGF1‐4, β‐tail, TM helix, and cytoplasmic tail domains. Integrin receptors can adopt a bent (left), an extended‐closed (center), and an extended‐open (right) conformation. They bind ligands typically at the cleft between the α‐ and β‐subunit heads (depicted in orange). (B) The cytoplasmic tails of β‐integrins can contain two different linear binding motifs (NPxY or NxxY) for various interaction partners (in boxes on the right) depending on β‐integrin isotype.

The main conductor of FAs, integrins, are type I transmembrane receptors that link the extracellular environment to the cytoskeleton of a cell (Figs 1 and 2). Integrins are heterodimers, consisting of a noncovalently linked α and β subunit (Fig. 2A). In vertebrates, 18 α and 8 β isoforms assemble into 24 different integrins, recognizing various ligands and exhibiting diverse expression profiles [5]. Integrins can be divided into four subclasses, depending on their extracellular ligand recognition profile, namely by binding to RGD, collagen, laminin, or leucocyte‐specific receptors [5]. Upon ligand binding, integrins undergo conformational changes that enable intracellular proteins to bind (Fig. 2B). Intracellular linker proteins such as talin and kindlin connect further to the actin cytoskeleton (Fig. 1) and transduce chemical and mechanical signals into the cell (outside‐in signaling). On the other hand, their ligand binding affinity can also be regulated by binding of intracellular proteins (inside‐out signaling). By changing their structural conformation from a bent‐closed to an extended‐open state [5], integrins can integrate and transduce stimuli across the plasma membrane, which affect downstream signaling and cell fate [4].

### Integrin–direct communicator between the outside and inside of a cell

Integrin α and β subunits are comprised of three main parts: the N‐terminal ligand‐binding ectodomain (~ 800 a.a.), a transmembrane (TM) helix (~ 20 a.a.), and the C‐terminal cytoplasmic tail (CT; ~ 13–79 a.a.) [19] (Fig. 2A). The ectodomains themselves are divided into head, upper leg, and lower leg domains [20]. The ligands are typically recognized by a cleft between the β‐propeller of the α subunit head and the βI domain of the β subunit head. The well‐studied RGD motif, a three amino acid sequence on many integrin ligands, such as fibronectin or fibrinogen, is recognized by both subunits [21, 22, 23]. Studies employing techniques such as X‐ray crystallography, electron microscopy, NMR, and light microscopy have revealed that integrins exist in three main conformations: bent, extended‐closed, and extended‐open (Fig. 2A) [5, 20, 22, 24, 25, 26, 27]. The head is in close proximity to the closed legs in the bent state with an exception of the recently reported integrin α5β1 showing an incomplete bent conformation [28]. In the bent conformation, integrin displays a low ligand binding affinity [20]. When extended, the integrin ectodomain is pointed away from the membrane [20]; the legs however are still closed (extended‐closed). Further opening of the integrin headpiece leads to the separation of the legs (extended‐open), and this conformation shows high ligand binding affinity. It has been suggested that integrins are constantly shifting between these different conformational states, which can be described as a conformational equilibrium or molecular breathing [5, 20]. The downstream TM domains of α‐ and β‐integrin form a coiled‐coil helix pair, securing the inactive form of integrin when closed [29, 30, 31, 32]. Upon opening of integrin, the separation of the coiled‐coil is thought to occur, accommodating a large conformational change of integrin [25, 29, 32]. The CT domains are relatively short, ranging from 13 to 79 amino acids in length, except for the β4 tail containing ~ 1000 amino acids. Despite their small size, integrin CT domains are considered as ‘interaction hubs’ for proteins of the intracellular signaling network [30, 33]. Particularly, the β CT domain is of importance, as it contains several recognition motifs for interaction partners, such as the NPxY and NxxY motifs (Fig. 2B) [19, 33]. The membrane‐proximal NPxY motif is recognized by phosphotyrosine binding (PTB)‐containing proteins, such as talin and DOK1 [34, 35] and, the membrane‐distal motif NxxY is recognized by proteins such as integrin cytoplasmic domain‐associated protein 1 (ICAP1), CD98, and kindlin [19, 30, 33, 36, 37, 38, 39, 40, 41].

Since multiple proteins can bind to partially overlapping regions on the β integrin CT domains and possibly compete with each other, the process of the protein interactions must be regulated [33]. Phosphorylation could be employed to switch between adaptor protein binding, as in the case of talin and DOK1 [42]. Similarly, the role of lipids in the recruitment of adaptor proteins has been suggested [43, 44, 45, 46]. Several adaptor proteins contain domains that are known for lipid binding (e.g., PH domain in kindlin) [33]. It has also been observed that increased lipid phosphorylation can take place within FAs by phosphatidylinositide 3‐kinase (PI3Ks) and phosphatidylinositol(4) phosphate 5 kinase type I gamma (PIPKI gamma) [47, 48, 49], which affects the affinity and recruitment of FA proteins like talin and kindlin to the plasma membrane.

### Intracellular focal adhesion adaptor proteins

**Fig. 3:**
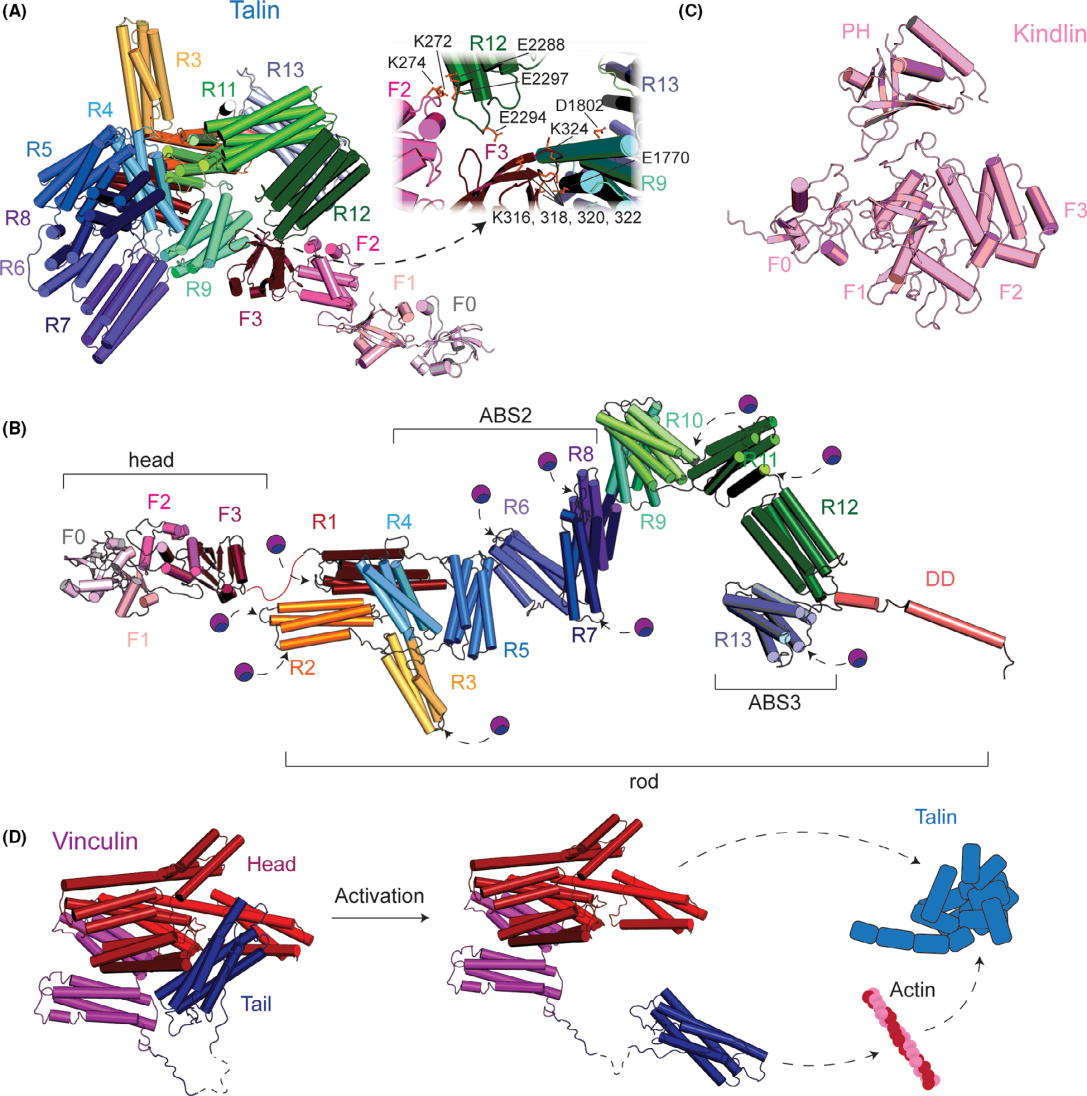
Domain architecture of talin, kindlin, and vinculin. (A) Talin consists of a globular FERM head (F0‐3) and a tail of 13 helical rod domains (R1–R13, in different colors) and a dimerization helix (DD, behind R13). In the autoinhibited form, all rod domains are entangled and the structure is secured by key interactions between F2–R12 and F3–R13 (insert). (B) Model of activated talin in an extended conformation with individual domains in different colors. Actin‐binding sites (ABS2 and ABS3) become accessible. Potential vinculin‐binding sites are highlighted with violet balls. (C) Kindlin consists of a globular FERM domain (F0–F3) and an additional PH domain. (D) Vinculin consists of a head domain comprising helical bundles D1–D4 and a helical tail that folds back in the autoinhibited state. The tail domain is released upon activation and opens binding sites for talin as well as for actin.

Out of the over 200 proteins involved in FA signaling, only a few of intracellular proteins are involved in the initial integrin activation and adhesion assembly steps. Talin and kindlin are two major FA components, which are exclusive integrin activators and key factors to trigger FA assembly. Both talin and kindlin contain 4.1‐ezrin‐radixin‐moesin (FERM) domains (Fig. 3) and bind to the CT domain of the β subunit of integrin (Fig. 2). Both proteins cooperate with each other and are needed to fully activate integrins [19, 50].

### Talin

Talin, as a main integrin activator, acts as a direct link between integrin and the actin cytoskeleton [50, 51]. As it is present in most integrin‐based adhesions and crucial for inside‐out activation of integrin, talin has been denoted as the core [52] or master of FAs [53]. Talin is also important for mechanotransduction, as it senses mechanical force and transduces it into biochemical signals [53]. In mammals, two isoforms of talin are present; talin‐1 is ubiquitously expressed and talin‐2 is mostly found in heart, brain, and kidney [52]. The significance of talin for proper cell adherence and function has been demonstrated by a plethora of studies. Knockout of talin leads to embryonic lethality in mice at E 8.5 [54] or severe defects when targeted to specific tissues [50]. On a cellular level, depleted talin can lead to decreased integrin activation and impaired cell spreading [55, 56, 57].

Talin is a 270‐kDa large protein consisting of an N‐terminal head followed by a rod domain connected by a long unstructured linker region (Fig. 3A,B). The talin head is an atypical FERM domain having an additional F0 domain. The rod domain is comprised of 62 α‐helices arranged into 13 helical bundles (R1–R13), consisting of 4–5 helices per bundle followed by a dimerization domain (DD) at the C terminus [50, 58].

## Regulation of talin

As talin is important for many cellular processes, several control and recruitment mechanisms tightly regulate its activation. *In vitro* studies showed that talin can exists either in a ~ 15‐nm globular, autoinhibited form or in a ~ 80‐nm extended conformation [53, 59, 60]. A recent cryo‐EM structure of autoinhibited talin displays how the long rod domains are folded in autoinhibited talin [60] (Fig. 3A), which occludes most major interaction sites to FA proteins such as actin, vinculin, and integrin. Two intramolecular interactions between the FERM domain and the rod domain, namely F3–R9 and F2–R12, were shown to be critical for autoinhibition. The F3–R9 connection shields the main integrin binding site 1 (IBS1) on F3 [45], while the F2–R12 connection shields the phosphatidylinositol‐4,5‐bisphosphate (PIP_2_) binding pocket on F2 [60] (Fig. 3A). The FERM domain F0–F1 is connected to F2 by a flexible linker and appears to be accessible when talin employs an autoinhibited conformation. This notion is also supported by the two crystal structures showing the arrangement of F0–F1 either as a linear extension from F2–F3 [61] or folding into a canonical compact form [62], reflecting its conformational dynamics. The recruitment of talin to membrane is suggested to be facilitated by the GTPase Rap1 [63]. Rap1 binds to the tip of F0, which is still accessible in the inhibited form of talin, which may suggest that talin may be recruited to the membrane surface in the autoinhibited form. Upon activation of talin through the engagement to FAs, the FERM domain may align linearly on the membrane surface in a tight interaction when PIP_2_ is present. Interestingly, full‐length talin was shown to act as a monomer in solution, while talin contains a short ~ 20 a.a. C‐terminal dimerization domain [58, 64] (Fig. 3B), which has been suggested to contribute to the cluster formation of FA components, necessary for FA maturation. Whether talin acts as a monomer or dimer when engaged in FAs is an important question to explore in future. For talin to act as a dimer, it may be necessary to have an interaction partner to align the extended talin for dimerization.

### Kindlin

The other known integrin activator is kindlin. It contains ~ 75 kDa F0–F3 FERM domain resembling the talin head, except for an additional inserted pleckstrin homology (PH) domain with affinity for PIP_2_ [19, 65] (Fig. 3b). In mammals, three isoforms exist: kindlin 1, 2, and 3, which exhibit varying expression patterns. Kindlin 1 is mainly expressed in epithelial cells and kindlin 3 in hematopoietic cells, while kindlin 2 is expressed ubiquitously, except for hematopoietic cells [19, 66]. Consistent with these expression profiles, knockout experiments in mice have shown that kindlin 1 deletion leads to severe defects in epithelial tissues, such as skin blistering [40]; kindlin 2 knockout leads to embryonic lethality [38] and deletion of kindlin 3 leads to severe bleeding disorders [41]. On a cellular level, kindlin depletion leads to impaired inside‐out integrin activation, FA formation and cell spreading, even when talin is present [38, 40, 41, 67]. Kindlins are reported to bind to the membrane‐distal NxxY motif of integrin β CT domains [19] (Fig. 2B). A crystal structure of kindlin 2 in complex with integrin β1 CT revealed a dimeric form of kindlin with an additional binding motif on the β tail, TTV, which is necessary for the recruitment of kindlin to FAs in cells [68]. Furthermore, a recent study showed a possibility that the autoinhibition of kindlin may be achieved through homotrimerization [69, 70], which blocks kindlin’s integrin binding site in the trimer structure [69]. While these studies implicated scenarios how kindlin is regulated, the exact role of kindlin, particularly whether oligomerization aids or inhibits integrin binding, is still an open question.

### Regulatory proteins of talin and kindlin

Talin, when elongated, can expose binding sites to a number of proteins [53, 60, 71]. Among the interactions provided by talin, particular importance lies in the connection between talin and actin as well as between talin, vinculin, and actin, as these complete the structural scaffolding of FAs. Besides the direct binding of talin to actin, vinculin plays a critical role for strengthening the connection by crosslinking talin and actin [72, 73, 74, 75, 76]. Vinculin is a 120 kDa globular protein containing 4 α‐helical bundles (D1–D4) in the talin‐binding head domain and another α‐helical bundle in its actin‐binding tail domain [77, 78] (Fig. 3D). In the autoinhibited conformation, they fold onto each other occluding binding sites to actin and talin (Fig. 3D). 11 potential vinculin binding sites are predicted along the talin sequence [79, 80] (Fig. 3B), and multiple vinculin binding on the talin surface may facilitate actin bundle formation in FAs. Truncated vinculin head, tail, and deregulated mutants have been used to circumvent the lack of understanding of the regulation of vinculin activation [81, 82, 83].

Other talin‐binding factors include RIAM (Rap1‐GTP‐interacting adaptor molecule) [64, 84], talin‐activator Kank [85], filamin [86], integrin cytoplasmic domain‐associated protein 1 (ICAP1) [37], DOK1 [34, 35], PI3PKγ [48, 49], and α‐actinin [87]. Each of these components interact not only with talin but also often with each other, facilitating to form an intricate network. However, how all these components are interplaying still has to be elucidated.

Kindlin also plays a role in transducing signals by interacting with several signaling proteins. Particularly, kindlin interacts with integrin‐linked kinase (ILK) [88], which then forms a complex with particularly interesting new cysteine‐histidine‐rich protein (PINCH) and parvin, forming the ILK‐PINCH‐parvin (IPP) complex. The IPP complex also plays a role in connecting integrin to the actin cytoskeleton (reviewed in [89]). Other kindlin interacting proteins include migfilin [90], paxillin [91], and actin‐related proteins 2/3 (Arp2/3) [92]. Paxillin binds to FAK and triggers Rho and Src signaling pathways that ultimately affect cell fate [93, 94].

## Bottom‐up analysis of the interplay within the FA initiation machinery

FAs undergo a complex network formation that contains multiple layers of regulation; therefore, it is challenging to elucidate the functions of individual factors at a molecular level. Many molecular studies have used truncated or deregulated proteins to focus on how distinct interactions of proteins of interest occur. However, using full‐length proteins and building up a machinery by a bottom‐up *in vitro* reconstitution approach gives valuable hints on their regulations. A particular interest lies in the initiation of the FA machinery. How exactly talin, kindlin, and other factors dock onto the integrin‐embedded plasma membrane surface and how they build up a machinery connecting to the actin cytoskeleton provides insight into the molecular basis of FAs.

### Integrin‐PIP_2_ containing membrane plus integrin activator talin and kindlin

As most of the FA proteins are tightly autoregulated, finding key to activate them is the critical first step for building up functional FA submachineries. Recent studies revealed that signaling lipid PIP_2_ plays an important role in the regulation and recruitment of the integrin activators talin and kindlin [61, 95, 96, 97, 98]. Our recent study additionally showed that autoinhibited full‐length talin binds to PIP_2_‐rich membrane surface, indicating that its autoinhibition is released upon binding to PIP_2_‐rich membrane [99].

**Fig. 4:**
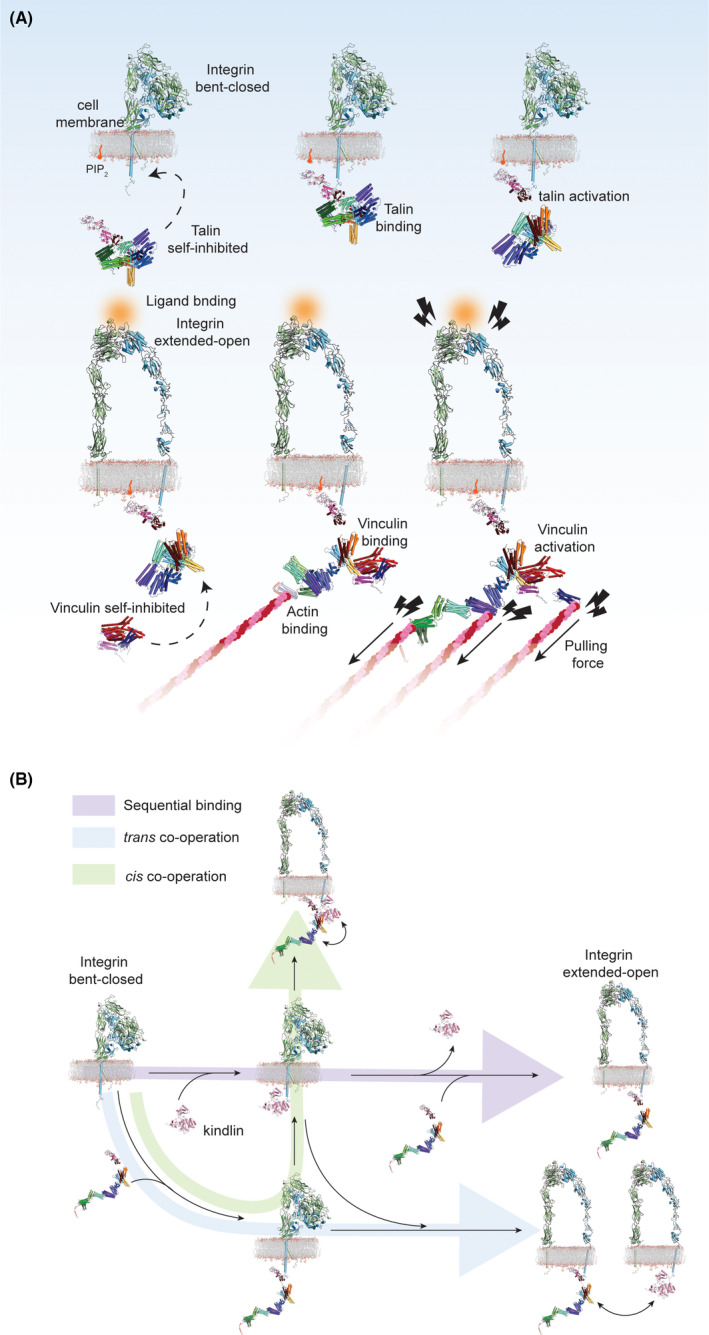
Schematic of integrin activation and FA initiation. (A) Autoinhibited talin can approach to the PIP2‐enriched membranes, resulting in the release of talin head and rod domains autoinhibition. Binding of talin to the cytoplasmic tails of β‐integrin primes the integrin receptor, which then binds to ligands in the extended‐open conformation. Opened talin can bind to actin, and this interaction is strengthened by crosslinking of talin and actin by activated vinculin. Further force‐dependent extension of talin (indicated by lightning bolts) uncovers additional vinculin binding sites. (B) Models of the cooperative activation of integrin by kindlin and talin. Integrin receptors can be activated by sequential binding of kindlin and talin (violet path), by simultaneous binding of both proteins to the same integrin receptor (cis cooperation, green path), or by synchronous binding of both proteins to different, clustered integrin receptors (trans cooperation, blue path).

By combining those with structural studies of the full‐length autoinhibited talin 1 [60] and the truncated talin 2 FERM domain in complex with the integrin β1D tail peptide [97], we can extrapolate our understanding on how activated talin then activates integrin on the membrane surface (Fig. 4A). When talin’s FERM domain is docked to the PIP_2_‐containing membrane surface, the tangled rod domain is likely detached from the FERM domain, which would expose the integrin binding surface on the F3 domain. Subsequently, the rod domain could flip away from the membrane so that it orients toward the cytosol. Membrane‐attached talin may readily recognize integrin because of its proximity to the plasma membrane as well as its exposure of the integrin binding site on the F3 domain. The exposed talin F3 domain binds to the NPxY motif in the integrin β CT. This interaction inhibits the salt bridge interaction between the integrin CT domains of the α and β subunits [34, 56, 97, 100, 101, 102, 103] inducing the separation of leg domains and the activation of integrin.

In contrast, it is still unclear how the regulation of kindlin and the involvement of PIP_2_ takes place. Kindlin might be necessary to cluster integrins [104] rather than opening the integrin α‐ and β‐CT domains like talin [105]. Future studies describing the dynamic process of kindlin and its interaction with talin, the PIP_2_ membrane as well as integrin are awaited to understand what the precise regulatory role of kindlin is. Nevertheless, neither talin nor kindlin alone are thought to be sufficient to activate integrins by themselves *in vivo*, and are thus considered as co‐dependent activators [19]. The binding mode remains elusive [19, 50] as it is not clear whether talin and kindlin interact directly on the same β‐tail (Fig. 4B). Other possibilities include that they bind sequentially to the same tail or simultaneously to different tails within the same integrin cluster (Fig. 4B) [19, 50]. It should however be noted that kindlin has been shown to be dispensable for integrin activation in an *in vitro* environment [18], and therefore, it may have rather an assisting role. Nevertheless, these are important questions that can be analyzed by *in vitro* reconstitution approaches.

### Recruitment of actin to the membrane surface

The docking of actin bundles to the FA initiation machinery located at the plasma membrane surface is a critical step for the development of FAs. That is mainly mediated by talin as it can directly bind to integrin, the plasma membrane through PIP_2_ and actin. Furthermore, vinculin strengthens the binding of talin and actin. These assemblies are the structural foundation of FAs. How actin can be recruited to the FA initiation site has been suggested through recent *in vitro* reconstitution studies [60, 99]. Those studies showed that autoinhibited talin is still accessible for actin through actin‐binding site 3 (ABS3) located at R13, while the other actin‐binding site ABS2 at R4–R8 is occluded and its actin‐binding function inhibited (Fig. 3A). ABS2 is ready to engage actin once the autoinhibition of talin is released by binding to PIP_2_‐enriched membranes (Figs. 3B and 4A). The opening of both ABS2 and ABS3 together facilitates binding of more actin filaments to talin. Upon binding of talin to integrin and actin, talin can extend to a 60‐ to 100‐nm‐long fibrous strand [11, 59, 106] (Fig. 4a). This elongation is thought to enable talin to span the distance between integrins and actin fibers [9] and to act as a cytoskeletal linker and mechanosensor [53]. At the same time, extended talin provides a platform for the binding of vinculin (Fig. 3B), reinforcing the engagement of actin to FAs. Interestingly, neither activated talin alone nor PIP_2_‐enriched membranes were shown to be sufficient to allow vinculin to bind to these components (Fig. 3D). It was however shown that activated talin attached to PIP_2_‐enriched membranes is able to activate vinculin and recruit it to the membrane surface where talin is localized [99] (Fig. 4A). While this is advantageous for the hierarchical regulation toward the engagement of actin bundles to the FA site, the precise molecular mechanism of action of vinculin regulation is still elusive.

### Mechanosensitive behavior of the FA components

As FAs provide a link between the ECM and cells, the molecules within FAs are constantly exposed to mechanical forces generated by both constant actin polymerization toward the membrane as well as actomyosin contraction of the cytoskeleton. Several of the components, such as integrin, talin, and vinculin, are found to be mechanosensitive [107, 108, 109, 110]. To characterize the force induced behavior of FA molecules, *in vitro* biophysical analyses have been instrumental. Increase of the strength of the integrin‐ECM bond with applied force was observed, both on isolated integrin as well as on cells [111, 112], which marks it as a so‐called catch‐bond [15]. The force that integrin‐ECM bonds can withstand until the bond breaks has been measured to be 50–100 pN [113]. In contrast, the conformation of talin is altered upon force, as it unfolds, which marks it as a slip‐bond [107]. The unfolding of talin leads to an exposure of cryptic binding sites for proteins like vinculin [79]. This suggested to expose some of the 11 cryptic vinculin binding sites, while only one vinculin binds to talin without applied force [60]. The recruitment of more vinculin to the stretched talin surface is suggested to promote FA growth [79, 80], but the precise mode of action of talin under force is still not well understood. Furthermore, the stretching of actin has been suggested to change the affinity of actin binding proteins such as vinculin [114]. Interestingly, the affinity of vinculin to actin depends on the direction of the applied force, implicating another layer of regulation to control vinculin binding at the FA site. As talin’s binding modules resemble the structural folding of vinculin, it is possible that talin may also be able to sense the stretching of actin, as for the case of catenin [115, 116, 117]. These examples hint at the regulation of several protein functions and the assembly of the FA initiation machinery by force. Taking the mechanosensitivity into consideration will therefore give more comprehensive insights into FA assembly.

## Maturation of FA and conclusion

The assembly process of FA complexes progresses in a highly coordinated and dynamic manner [51, 118]. Upon generation of a positive feedback loop, which is directly linked to mechanical force, that is, ECM stiffness and myosin II contractility [17, 119], the maturation of FAs is thought to occur where hundreds of FA components start forming an interconnected network. Building up the FA machinery from minimal components gives a great advantage for elucidating key interactions within FAs and provides a hierarchical understanding of the molecular actions of individual players. That provides a basis for the understanding of the layers of the vast FA network formation.

## Conflict of interest

The authors declare no conflicts of interest.

## Author contributions

SS, CB., and NM collected the relevant literature and wrote the review manuscript. RVN prepared the figures. All the authors read and approved the submitted version.
